# *Iranocichla persa*, a new cichlid species from southern Iran (Teleostei, Cichlidae)

**DOI:** 10.3897/zookeys.636.10571

**Published:** 2016-11-24

**Authors:** Hamid Reza Esmaeili, Golnaz Sayyadzadeh, Ole Seehausen

**Affiliations:** 1 Ichthyology and Molecular Systematics Lab., Department of Biology, College of Sciences, Shiraz University, Shiraz, Iran; 2 Department of Fish Ecology & Evolution, EAWAG Centre for Ecology, Evolution and Biogeochemistry, 6047 Kastanienbaum, & Division of Aquatic Ecology, Institute of Ecology and Evolution, University of Bern, 3012 Bern, Switzerland

**Keywords:** Barcode region, inland fish, Middle East, Persian Gulf

## Abstract

*Iranocichla persa* **sp. n.** is described from the Shur, Hasanlangi and Minab River drainages flowing into the Persian Gulf at the Strait of Hormuz in southern Iran. It is distinguished from *Iranocichla hormuzensis*, from the Mehran River drainage, by nuptial males having a bright orange breast and lower part of the head (vs. black), a poorly developed or invisible (vs. distinctive) “*Tilapia*-mark” in the dorsal fin and very clear white spots making almost wavy bars or stripes on the caudal fin (vs. without or with very few white spots). Mitochondrial DNA sequence characters suggest that both *Iranocichla* species are closely related but form two distinct clades, diagnosable by several fixed mutations in ND2, D-loop and partially by COI sequences. Populations from Kol River drainage, which is situated in-between the Mehran and the Shur River drainages, are more similar to *Iranocichla hormuzensis* in terms of their male nuptial coloration but to *Iranocichla persa* **sp. n.** in their mitochondrial sequence characters. Their status requires further investigation.

## Introduction

The presence of a cichlid species in southern Iran was first noted by Behnke (1975) and these fishes were briefly described but not named by Saadati (1977) in his MS thesis. Coad (1982) described the Iranian cichlids as a new genus and species, *Iranocichla hormuzensis*, based on fishes from the Mehran River drainage and it was considered as the only cichlid fish of Iran (Esmaeili et al. 2010, 2015). In 2013, HRE collected 82 *Iranocichla* individuals from all over its range. Schwarzer et al. (2016) analysed all these 82 individuals for their life coloration and 75 of them for their mitochondrial ND2 and D-loop sequences (2044 bp). Schwarzer et al. (2016) suggest the presence of three phenotypically distinct phylogeographic groups in *Iranocichla*. Fishes from the Mehran River drainage, which is the westernmost river inhabited by *Iranocichla*, form one of these clades corresponding to *Iranocichla hormuzensis*. Fishes from the Shur, Hasanlangi and Minab River drainages, in the eastern part of the range of *Iranocichla*, correspond to the majority of individuals of a second clade identified by Schwarzer et al. (2016), here described as *Iranocichla persa*. The third group identified by Schwarzer et al. (2016) inhabits the Kol River drainage, which is situated between the Mehran and Shur Rivers. Most of these fish form a third mitochondrial clade, closely related to the *Iranocichla persa* clade but some are part of the latter clade. Hence, this third group appears polyphyletic, albeit only a single haplotype in a single individual was shared with *Iranocichla persa*. Nuptial males from all three genetic groups can be differentiated by their coloration. Nuptial males of *Iranocichla hormuzensis* show a blue or black breast and head while those from the Shur, Hasanlangi and Minab have a bright orange breast and lower part of the head, a trait not known from any other Oreochromine cichlid. Nuptial males of the third molecular group resemble *Iranocichla hormuzensis* in these traits, although they differ from them in other coloration traits (see below), but are closer to *Iranocichla persa* in their mitochondrial sequences. Based on mitochondrial D-loop and ND2 datasets, the reciprocal monophyly of Mehran River populations (clade A) and of Shur, Rudan and Kol populations (clade B) was supported in Schwarzer et al. (2016). This is confirmed here with a set of mitochondrial cytochrome c oxidase subunit 1 (COI) sequences. These findings are discussed and the populations with an orange breast and head in nuptial males are described as a new species.

## Material and methods

**Figure 1.**
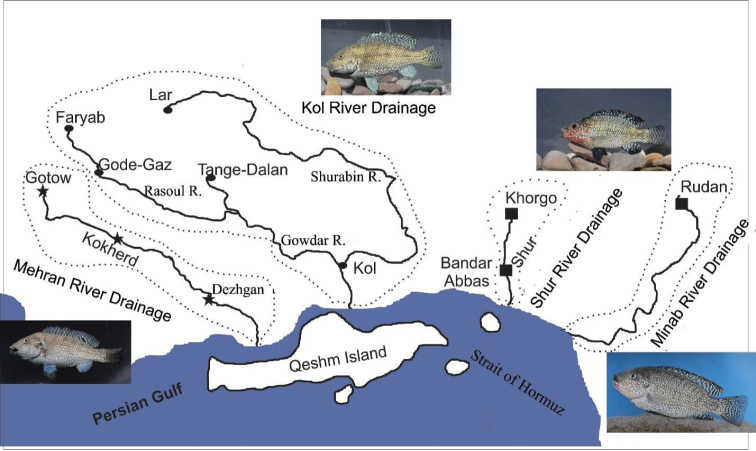
Geographic distribution map of *Iranocichla* populations in four river drainages of Iran. Symbols indicate our sampling sites and the different taxa. Asterisk = *Iranocichla hormuzensis*, rectangle = *Iranocichla persa* sp. n., circle = *Iranocichla* sp. “Kol”.

To study nuptial coloration, all fishes across the distribution range of the genus were collected during their reproductive season in March 2013 using cast and hand nets (Fig. 1). Nuptial males were photographed alive in a portable aquarium, immediately after being captured. After anaesthesia, fishes were either fixed in 10% formaldehyde or in 70% ethanol and all fishes were later transferred to and stored in 70% ethanol. Measurements were made with a dial caliper and recorded to 0.1 mm. All measurements were made point to point, never by projections. Methods for counts and measurements follow Coad (1982). Standard length (SL) was measured from the tip of the snout to the end of the hypural complex. The length of the caudal peduncle was measured from the insertion of the last anal-fin ray to the end of the hypural complex at mid-height of the caudal-fin base. The holotype is included in the calculation of means and SD. Abbreviations used: SL, standard length; HL, head length; FSJF, Fischsammlung J. Freyhof, Berlin; ZM-CBSU, Zoological Museum of Shiraz University, Collection of Biology Department, Shiraz.

**DNA extraction and PCR.** Genomic DNA was extracted using Macherey and Nagel NucleoSpin® Tissue kits following the manufacturer’s protocol on an Eppendorf EpMotion® pipetting-roboter with vacuum manifold. The standard vertebrate DNA barcode region of the COI (cytochrome c oxidase subunit 1) was amplified using a M13 tailed primer cocktail including FishF2_t1 (5’TGTAAAACGACGGCCAGTCGACTAATCATAAAGATATCGGCAC), FishR2_t1 (5’CAGGAAACAGCTATGACACTTCAGGGTGACCGAAGAATCAGAA), VF2_t1 (5’TGTAAAACGACGGCCAGTCAACCAACCACAAAGACATTGGCAC) and FR1d_t1 (5’CAGGAAACAGCTATGACACCTCAGGGTGTCCGAARAAYCARAA) (Ivanova et al. 2007). Sequencing of the ExoSAP-IT (USB) purified PCR product in both directions was conducted at Macrogen Europe Laboratories with forward sequencing primer M13F (5’GTAAAACGACGGCCAGT) and reverse sequencing primer M13R-pUC (5’CAGGAAACAGCTATGAC).

**Molecular data analysis.** Data processing and sequence assembly was done in Geneious (Biomatters 2013) and the Muscle algorithm (Edgar 2004) chosen to create a DNA sequence alignment. Modeltest (Posada and Crandall 1998), implemented in the MEGA 6 software (Tamura et al. 2013), was used to determine the most appropriate sequence evolution model for the given data, treating gaps and missing data with the partial deletion option under 95% site coverage cutoff. The model with the lowest BIC scores (Bayesian Information Criterion) is considered to best describe the substitution pattern. According to Modeltest, the Tamura-Nei model (Tamura and Nei 1993) with discrete Gamma distribution (5 categories (+G, parameter = 0.4292)), best represented the COI alignment, and was used to estimate the evolutionary history.

Maximum Likelihood (ML) phylogenetic trees were generated with 10,000 bootstrap replicates in RaxML software 7.2.5 (Stamatakis 2006) under the GTR+G+I model of nucleotide substitution, with CAT approximation of rate heterogeneity and fast bootstrap to explore species phylogenetic affinities. Bayesian analyses of nucleotide sequences were run with the parallel version of MrBayes 3.1.2 (Ronquist and Huelsenbeck 2003) on a Linux cluster with one processor assigned to each Markov chain under the most generalizing model (GTR+G+I) because over-parametrization apparently does not negatively affect Bayesian analyses (Huelsenbeck and Ranala 2004). Each Bayesian analysis comprised two simultaneous runs of four Metropolis-coupled Markov-chains at the default temperature (0.2). Analyses were terminated after the chains converged significantly, as indicated by the average standard deviation of split frequencies <0.01. Bayesian inference of phylogeny was conducted for 6,000,000 generations.

## Results

**Figure 2.**
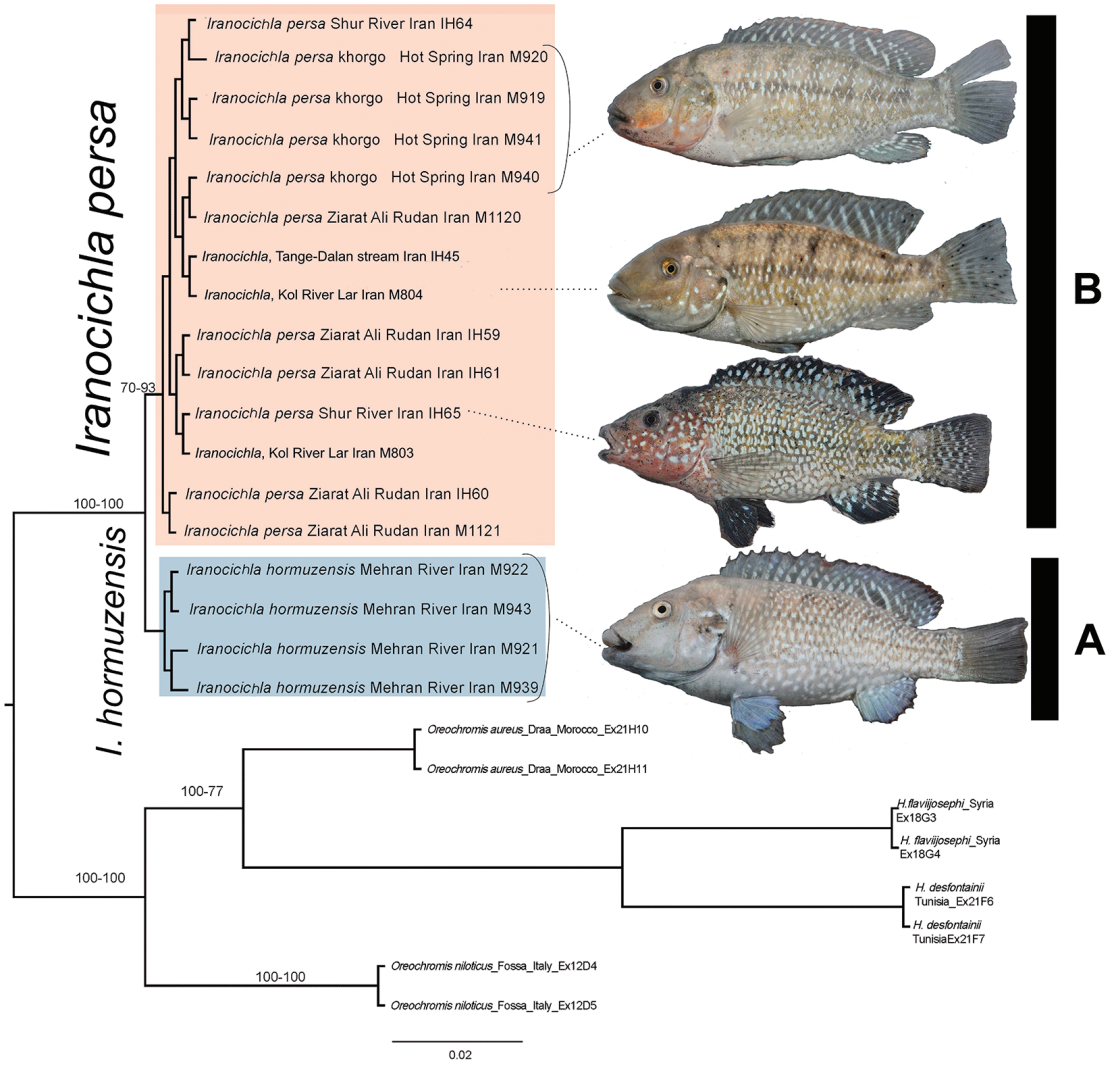
Maximum Likelihood estimation of the phylogenetic relationships of *Iranocichla* species based on the mitochondrial COI barcode region. Nucleotide positions with less than 95% site coverage were eliminated before analysis. Numbers of major nodes indicate bootstrap values from the Maximum Likelihood-method from 1000 pseudo-replicates, followed by Bayesian posterior probabilities.

COI barcode sequences are included for a total of 18 individuals of *Iranocichla* from its distribution range over four different river drainages (Mehran, Kol, Shur and Minab). Maximum Likelihood-based estimation of the phylogenetic relationships based on the mitochondrial COI barcode region placed the sequenced *Iranocichla* individuals into two closely related groups (Fig. 2). The four individuals from the Mehran River form one clade and the 14 individuals from the Kol, Shur and Rudan River drainages formed a second clade, fully consistent with the published data from other mitochondrial genes (Schwarzer et al. 2016).

### Iranocichla persa sp. n.

Taxon classificationAnimaliaPerciformesCichlidae

http://zoobank.org/B2A4CC63-A989-41F3-8E61-245E4B373B45

Figs 3 , 4 , 5

#### Holotype.

ZM-CBSU IP66, 89 mm SL; Iran: Hormuzgan prov.: Shur River approx. 30 km east of Bandar Abbas, 27°17'40.10"N 56°29'15.68"E; H. R. Esmaeili, M. Masoudi, H. Mehraban, A. Gholamifard & N. Shabani, 12 March 2013.

#### Paratypes.

All from Iran: Hormuzgan prov.: ZM-CBSU IP64, 2, 65-87 mm SL, same data as holotype. ZM-CBSU IP67-ZM-CBSU K1120, 20, 65-86 mm SL; Khorgo (Khorgu) hot spring approx. 50 km north east of Bandar Abbas, 27°31'21.3"N 56°28'12.7"E; H. R. Esmaeili, M. Masoudi, H. Mehraban, A. Gholamifard & N. Shabani, 12 March 2013. — ZM-CBSU IP59, 5, 74-88 mm SL; Rudan river at Ziarat Ali village, approx. 30 km north of Rudan, 27°45'44.42"N 57°14'34.33"E; H. R. Esmaeili, M. Masoudi, H. Mehraban, A. Gholamifard & Shabani, 11 March 2013. —ZM-CBSU IP141, 5, 82-102 mm SL; Rudan river at Ziarat Ali village, approx. 30 km north of Rudan, 27°45'44.42"N 57°14'34.33"E; M. Masoudi & H. Mehraban, 9 April 2014. —FSJF 3468, 63-81 mm SL; Khorgo Hot spring approx. 50 km north east of Bandar Abbas, 27°31'21.3"N 56°28'12.7"E; H. R. Esmaeili, M. Masoudi, H. Mehraban, A. Gholamifard & N. Shabani, 12 March 2013.

#### Materials used for molecular analysis.

All from Iran: Hormuzgan prov.: ZM-CBSU M919, M920, M940, M941; Khorgo hot spring, 27°31'21.3"N 56°28'12.7"E (GenBank accession numbers: KY034435, KY034436, KY034437, KY034438). —ZM-CBSU M1120, M1121, ZM-CBSU-IH59, ZM-CBSU-IH60, ZM-CBSU-IH61; Rudan River at Ziarat Ali village, 27°45'44.42"N 57°14'34.33"E (GenBank accession numbers: KY034442, KY034443, KY034439, KY034440, KY034441). —ZM-CBSU-IH64, ZM-CBSU-IH65; Shur River, 27°17'40.10"N 56°29'15.68"E (GenBank accession numbers: KY034444, KY034445).

#### Diagnosis.

*Iranocichla persa* is distinguished from *Iranocichla hormuzensis* by its nuptial coloration in males. In *Iranocichla persa*, the lower part of the head and breast are orange (vs. black), the background colour of the flank is grey with an orange hue (vs. black), each scale is furnished with an iridescent patch and these patches take up more space (vs. less) than the space between them, a poorly developed or invisible (vs. distinctive) “*Tilapia*-mark” in the dorsal fin, and very clear white spots making almost wavy bars or stripes on the caudal fin (vs. without or with very few white spots). Both species are also distinguished by multiple fixed molecular characters in mitochondrial ND2, D-loop (see Schwarzer et al. 2016).

#### Description.

**Figure 3.**
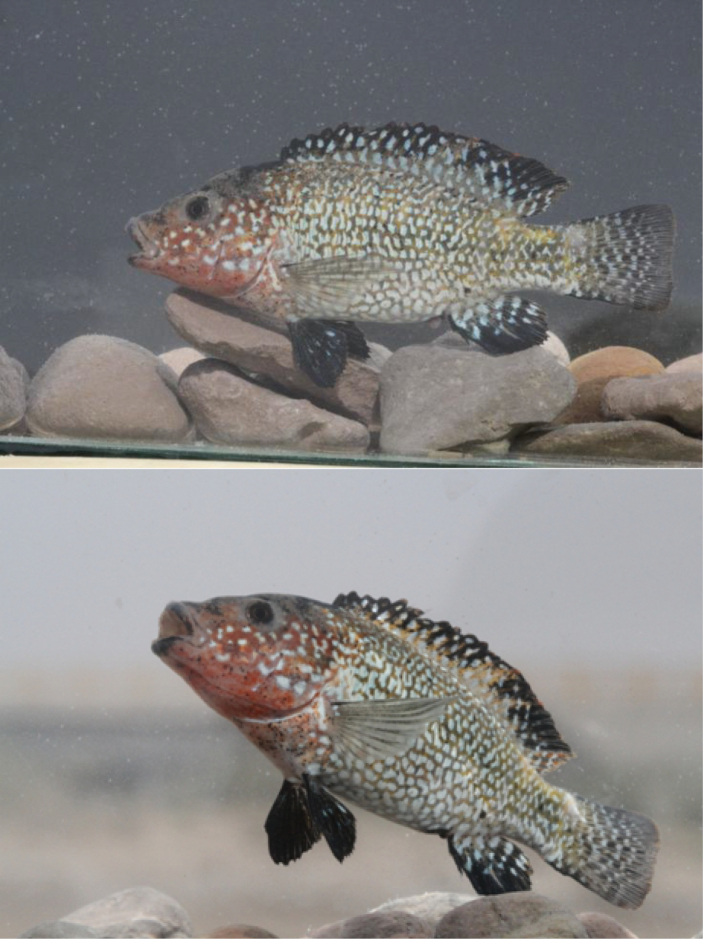
*Iranocichla persa*, ZM-CBSU-IP66, male, holotype, 89.54 mm SL; Hormuzgan prov.: Shur River.

**Figure 4.**
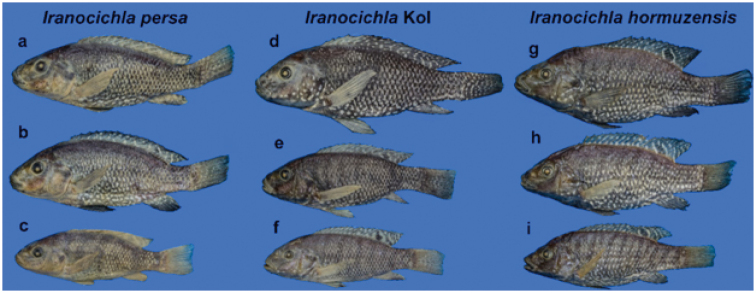
Males of the three *Iranocichla* taxa from Hormuzgan prov., Iran: *Iranocichla persa*; paratypes; **a** ZM-CBSU-IP75, 86 mm SL **b** ZM-CBSU-IP78, 82 mm SL **c** ZM-CBSU-IP69, 67 mm SL; Khorgo Hot spring. *Iranocichla* from Kol **d** ZM-CBSU-IP34, 113 mm SL **e** ZM-CBSU-IP38, 84 mm SL **f** ZM-CBSU-IH45, 78 mm SL; Kol River drainage. *Iranocichla hormuzensis* **g** ZM-CBSU-IH55, 90 mm SL **h** ZM-CBSU-IH51, 83 mm SL **i** ZM-CBSU-IH49, 72.2 mm SL; Mehran River drainage.

**Figure 5.**
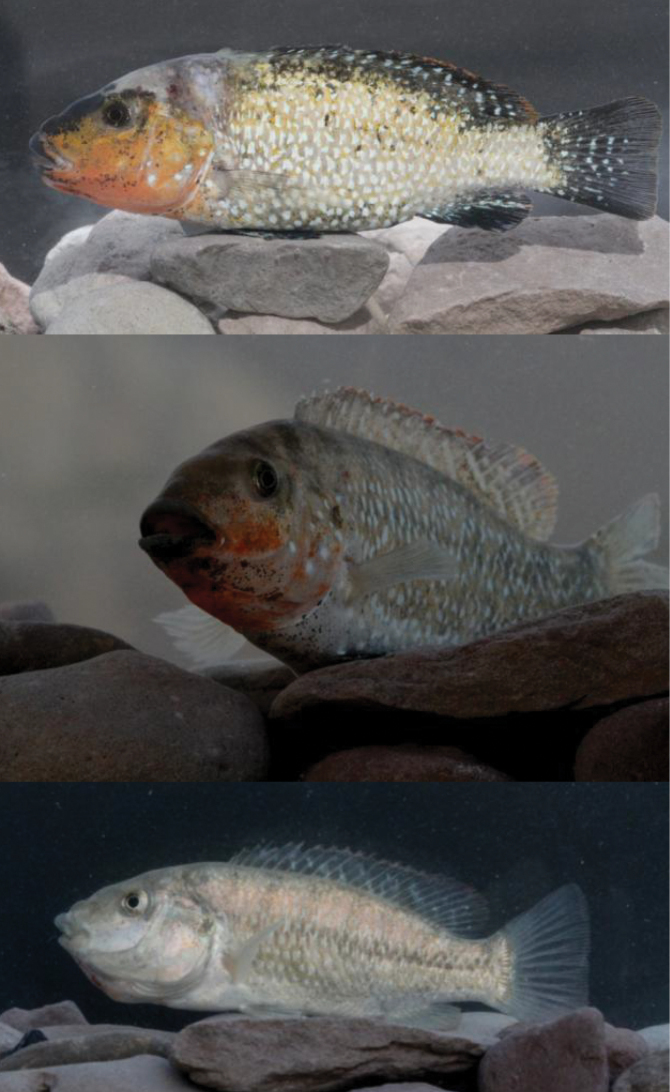
*Iranocichla persa*, ZM-CBSU-IP64, male, paratype, 86.7 mm SL; Hormuzgan prov.: Shur River, ZM-CBSU-IP67, male, paratype, 75.8 mm SL; Hormuzgan prov.: Khorgo Hot spring, ZM-CBSU-IP73, female with eggs in her mouth, paratype, 76 mm SL; Hormuzgan prov.: Khorgo Hot spring.

See Figure 3–5 for general appearance. Morphometric data are provided in Table 1. A small species with greatest body depth at approximately fifth dorsal-fin spine. Dorsal body profile convex from anterior part of dorsal fin to caudal peduncle. Ventral body profile straight or slightly convex between pelvic and anal fins. Dorsal head profile straight, slightly concave between nostrils and interorbital space. Head and eyes large. Mouth terminal, tip of upper and lower jaws at same vertical line (isognathous). Upper lip noticeably thickened, buccal region enlarged ventrally, oral teeth uniform in size and not enlarged medially.

**Table 1.** Morphometric and meristic data of *Iranocichla persa*, (holotype, ZM-CBSU-IP66; paratypes, ZM-CBSU-IP59-IP65, IP67-IP78, K1120-K1127, n = 33).

	males (n = 18)				females (n = 15)			
	Min	Max	Mean	SD	Min	Max	Mean	SD
Standard length (mm)	66.6	102	82.5	8.5	63.7	88.7	73.0	7.7
In percentage of standard length								
Head length	33.8	37.0	34.9	0.9	33.5	37.6	36.1	1.0
Pre dorsal length	34.5	38.9	36.6	1.2	34.7	40.3	38.3	1.5
Post dorsal length	32.3	37.0	34.3	1.2	31.7	35.4	33.8	1.0
Dorsal fin length	49.6	53.7	51.4	1.1	46.3	52.0	49.3	1.7
Anal fin length	10.5	12.7	11.4	0.7	9.0	12.1	10.7	0.8
Pre-anal length	66.8	72.4	70.4	1.2	70.1	73.9	72.3	1.2
Pectoral fin length	21.4	29.4	25.7	2.1	23.0	27.1	24.7	1.2
Pelvic fin length	17.4	22.6	19.5	1.6	16.3	20.2	17.9	1.2
Pre-pelvic length	35.4	39.7	38.0	1.1	37.3	41.6	39.9	1.3
Maximum body depth	32.7	37.0	34.7	1.2	30.9	37.9	34.2	2.4
Body depth at dorsal fin origin	30.4	35.2	32.7	1.5	30.9	34.5	32.4	1.2
Minimum body depth	11.8	14.0	12.9	0.6	11.8	13.8	12.7	0.7
Distance between P&V	11.4	14.2	13.1	0.7	11.5	15.5	13.3	1.2
Distance between V&A	32.5	37.3	34.3	1.3	31.9	36.4	33.7	1.4
Caudal fin length	21.7	26.1	23.9	1.3	21.9	25.4	23.3	1.1
Caudal peduncle length	18.1	24.7	20.5	1.5	17.3	20.7	19.3	0.9
In percentage of head length								
Head depth	64.5	83.2	73.9	5.2	64.4	79.1	69.5	4.9
Head width	48.5	57.7	53.0	2.7	48.7	57.2	53.4	2.6
Preorbital distance	37.2	44.6	41.0	1.9	35.8	45.8	41.6	2.1
Postorbital distance	42.8	50.8	44.2	1.8	42.2	46.5	44.4	1.4
Interorbital distance	26.9	33.0	29.2	2.0	25.8	39.3	31.3	3.3
Eye diameter	16.7	20.8	18.8	1.1	16.5	20.2	17.9	1.1
Meristic characters								
Scales in upper lateral line	17	24	19.9	1.9	17	22	19.3	1.9
Scales in lower lateral line	9	13	11.2	1.4	9.0	13.0	10.4	1.2
Dorsal fin unbranched rays	14	17	15.4	0.8	14.0	17.0	15.3	0.8
Dorsal fin branched rays	9	10	9.7	0.5	9	10	9.6	0.5
Anal fin unbranched rays	3	3	3.0	0.0	3	3.0	3	0.0
Anal fin branched rays	6	8	6.7	0.6	6	7	6.7	0.4
Pelvic fin unbranched rays	1	1	1.0	0.0	1	1	1.0	0.0
Pelvic fin branched rays	5	55	5	0	5	5	5	0
Pectoral fin rays	12	12	12.0	0.0	11	12	11.9	0.2
Gill rakers	14	17	15.4	0.7	14	17.	15.1	1.0

Dorsal-fin base long, its origin at a vertical of pectoral-fin base, base of last dorsal-fin ray at vertical of posterior part of anal-fin base. Posterior dorsal-fin tip reaching to a point slightly in front of caudal-fin origin when folded back. Dorsal fin with 14–17 spines and 9½-10½ branched rays. Anal fin with 3 spines and 6½-8½ branched rays. Pelvic fin with 1 spine and 5 branched rays, not reaching to anus. Pectoral fin long with 11–12 branched rays, third branched ray being longest, reaching to vertical of 9^th^-11^th^ dorsal-fin spine. Caudal fin truncate or slightly emarginated with 8+8 or 9+8 branched rays. Upper lateral line with 17–24 pored scales, starting from posterior tip of operculum to a vertical of 3^rd^-4^th^ branched dorsal-fin ray. Lower lateral line with 9–13 pored scales, reaching from a vertical of 3^rd^-4^th^ branched dorsal fin rays to caudal-fin base. Scales cycloid or having very small ctenius-like structure, regularly arranged on flanks except that in a few larger individuals (≥85 mm SL; 3 out of 9 specimens), where scale rows are interspaced by irregularly set smaller scales, particularly on the upper flank. Head without scales in some individuals, dorsal and anal fin bases without scales, no scale between the pectoral and pelvic fin bases and none on the belly and isthmus anterior to the pelvic fin. Upper margin of operculum without scales or with 1–2 large scales next to each other and subopercular bone without scales or with one scale at middle. Cheek without scales or with 1–3 rows of 1–7 almost non-imbricate scales. 11–12 rows of small scales on caudal-fin base, extending distally along more than half of the fin ray length in some individuals and extending distally along equal or less than half in some others.

**Figure 6.**
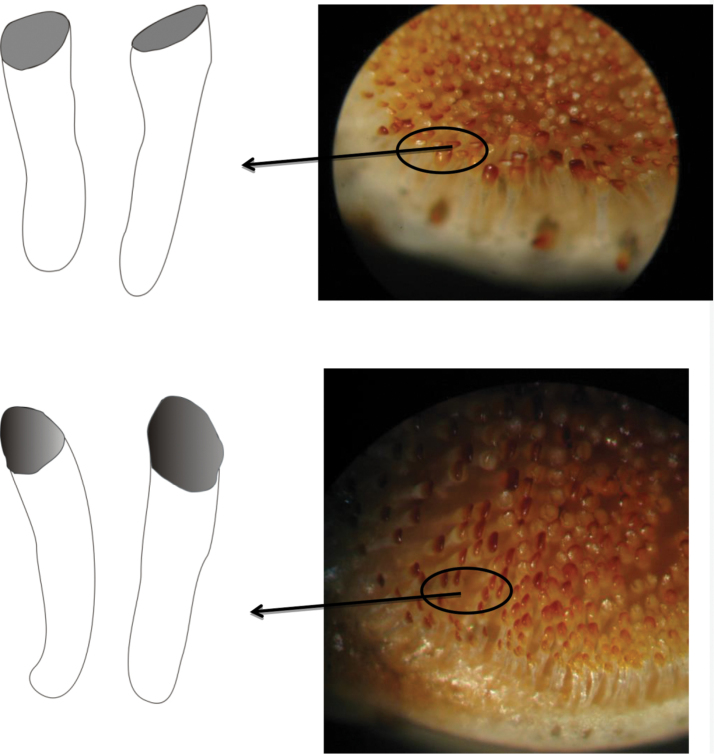
Lower and upper pharyngeal teeth of *Iranocichla persa*.

**Figure 7.**
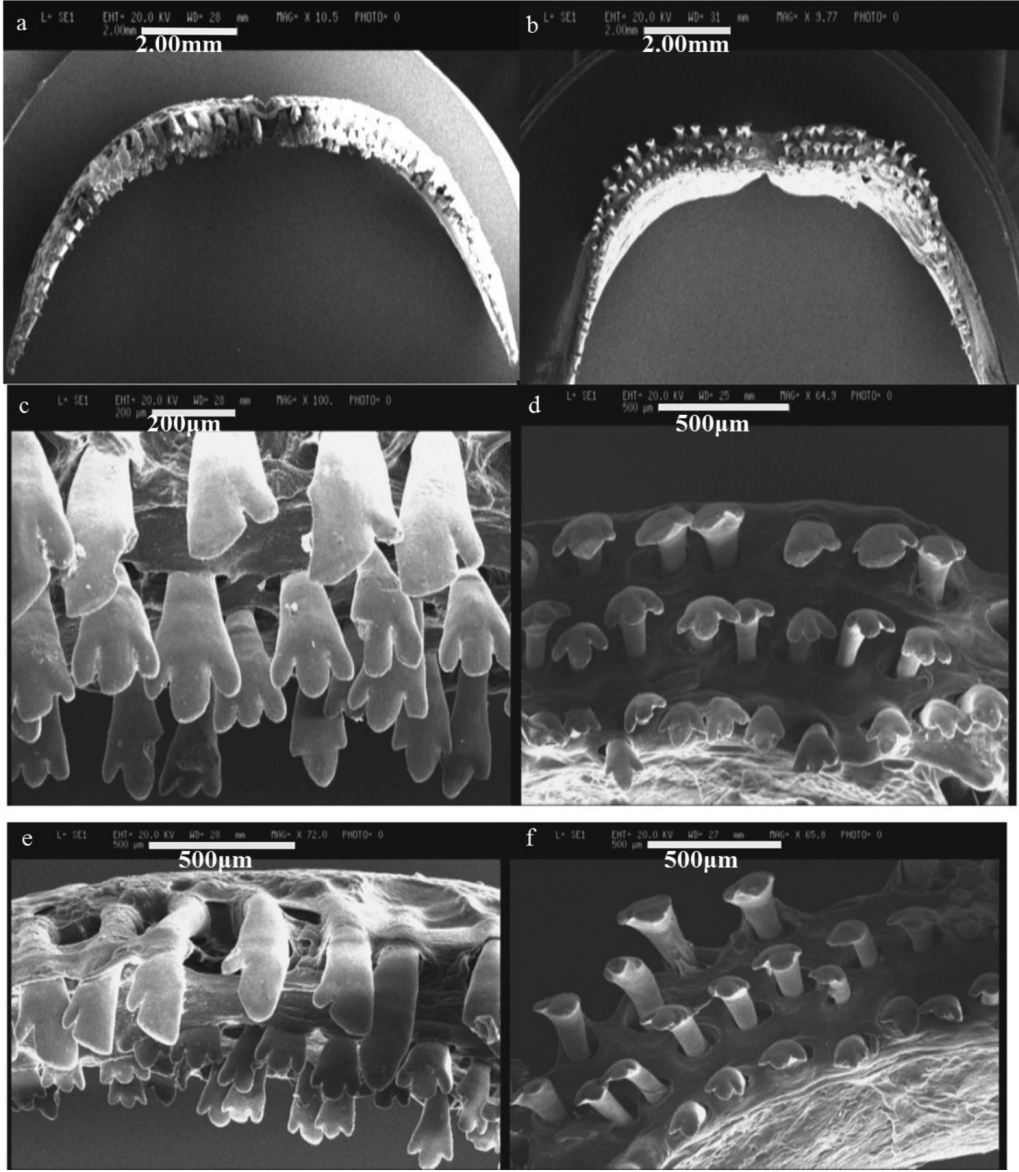
SEM photos of jaw teeth of *Iranocichla persa*; **a, c, e** upper jaw **b, d, f** lower jaw.

**Figure 8.**
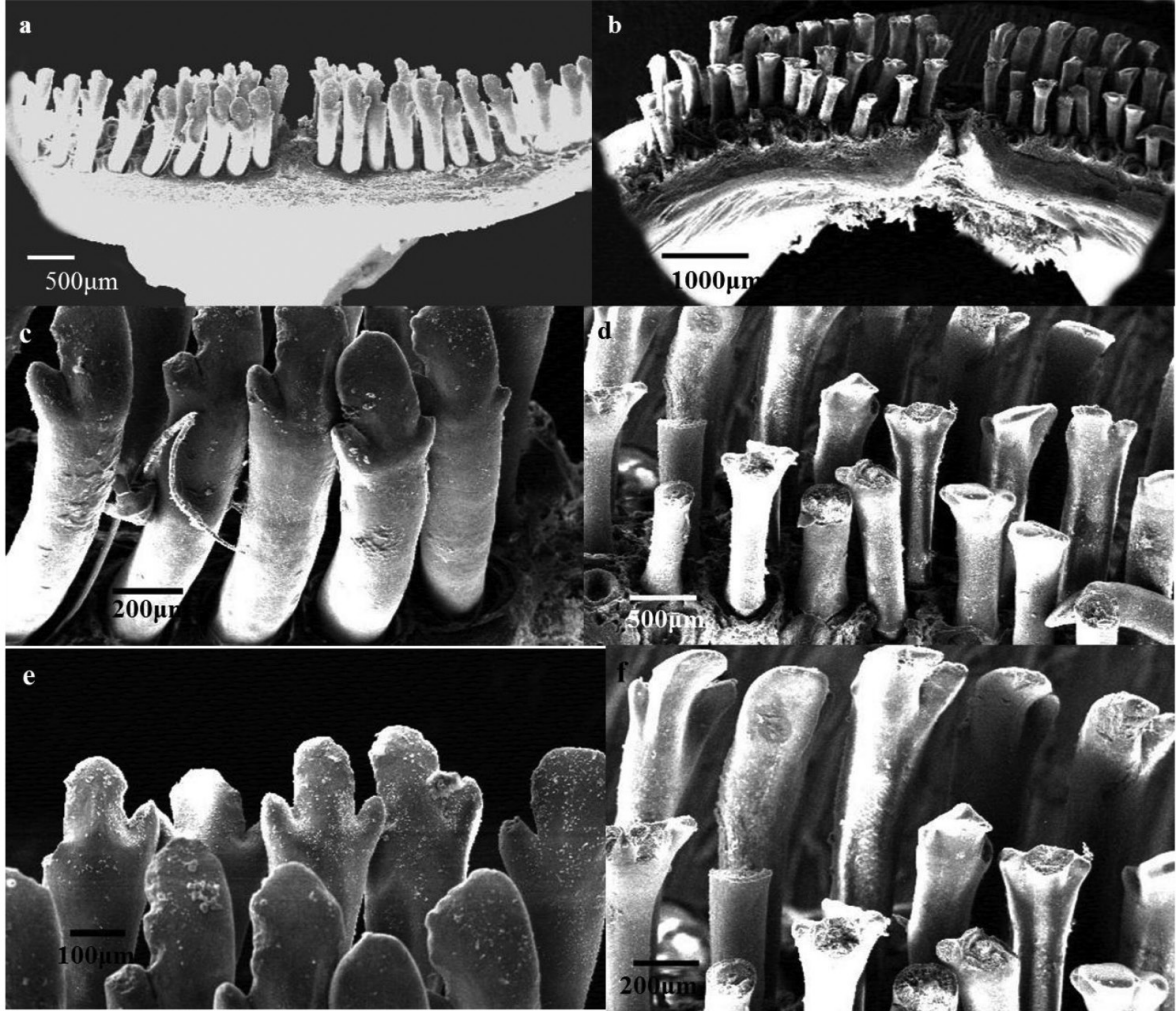
SEM photos of Jaws teeth of *Iranocichla hormuzensis*; **a, c, e** upper jaw **b, d, f** lower jaw.

Teeth in oral jaws regularly or irregularly arranged, 3-4 rows in both jaws (of the four examined, two individuals with 3 rows in upper and 4 in lower jaw). Number of rows decreases laterally to one row at rictus. Teeth in outer row widely spaced, spaces often nearly as wide as the crown, mostly bicuspid, major cusp with a protracted flank, but a few teeth tricuspid. Teeth in inner row tricuspid, central cusp largest (see Figs 6–8).

#### Sexual dimorphism.

Nuptial males with an orange breast and lower part of head and few roundish white spots on cheek and operculum. Females have a longer head on average (33–38% SL vs. 34–37% SL), a wider interorbital distance (26–39% HL vs. 27–33% HL) and shorter pelvic fin (16–20% SL vs. 17–23% SL) as compared to males.

#### Colouration.

In life. Background colour silvery grey or yellowish, a dark grey narrow saddle between eyes and a dark grey band at nape between uppermost parts of operculum. A dark grey, faint mid-lateral stripe between posterior eye margin and caudal-fin base and a second, often indistinct, dorso-lateral stripe between nape and “*Tilapia* mark”. Dorso-lateral stripe often dissociated into a marbled pattern. Mid-lateral stripe often dissociated into a series of vertically elongated large blotches at intersection with vertical bars. Body with 6–11 (mode 8) faint, wide, vertical bars, first bar at level of third dorsal-fin spine, last bar on posterior-most caudal peduncle. Bars most prominent above midlateral line, faded below. Bars almost or fully absent in nuptial males. Dorsal fin hyaline or grey with black “*Tilapia* mark” on posterior part of dorsal fin (absent in nuptial males). Caudal, anal, pelvic and pectoral fins grey or hyaline. Caudal fin with a series of 5–6 narrow vertical bars in some males, uniformly grey in other males and in all females.

Nuptial males with a prominent orange hue on flank. “*Tilapia* mark” absent. Lower head to upper eye margin orange, in some individuals with very small dark brown spots. Roundish white iridescent spots on cheek and operculum, Breast pale or orange. Breast and belly with very small dark brown spots in some individuals. Forehead and nape black in some individuals. Lips black at outer margin and orange at inner margin. Body except breast and nape with a prominent iridescent spot or small blotch on each scale. White blotches narrow, comma shaped, vertically elongated, most prominent on or restricted to posterior scale margins on upper flank above a horizontal line between pectoral-fin origin and posterior anal-fin base or a bit above that line. Below that line, iridescent spots and blotches on posterior scale margin roundish or ovoid, often irregularly shaped. On caudal peduncle and body behind a vertical line between last dorsal-fin spine and anal-fin origin, white spots narrow, restricted to posterior scale margin or along complete free scale margin, forming a reticulate pattern on caudal peduncle. Some individuals with irregularly x-shaped white blotches on anterior flank, roundish or ovoid on belly and comma-shaped, short vermiculate or roundish on dorsal and posterior flank. Dorsal fin with orange margin in most nuptial males, black in others. Dorsal fin rays hyaline, grey or black. Spines, membranes with white roundish or vertically elongated blotches, some fused to forward slanted narrow bars. Caudal fin grey or black with very clear white spots making almost wavy bars or stripes on the caudal fin. Anal fin grey with black distal anterior edge, with a few white, roundish, elongate or comma shaped blotches, most prominent on proximal and posterior parts of anal fin, absent on distal and anterior parts. Pelvic fin grey, light blue or black with few or no white spots or blotches. Pectoral fin hyaline or with black rays.

#### Distribution.

**Figure 9.**
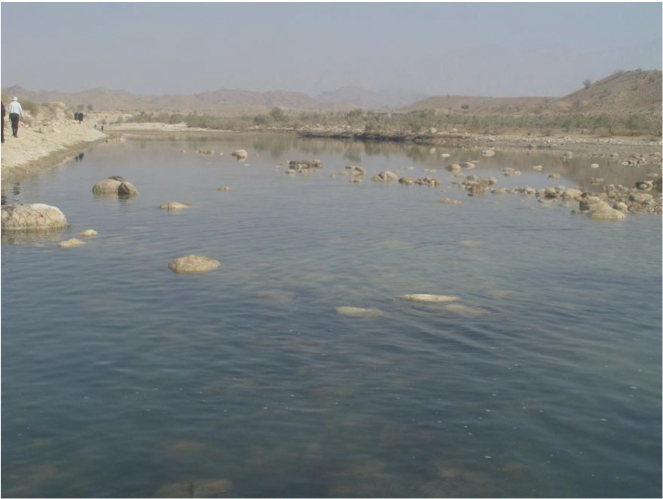
Habitat of *Iranocichla persa*, Khorgo hot spring, Shur River drainage, Iran.

*Iranocichla persa* is known from the Shur (Fig. 9), Hasanlangi and Minab River drainages flowing to the Persian Gulf at the Strait of Hormuz (Fig. 1).

#### Etymology.

The species is named for Persia, the ancient name of Iran.

#### Remarks on populations from the Kol River drainage.

**Figure 10.**
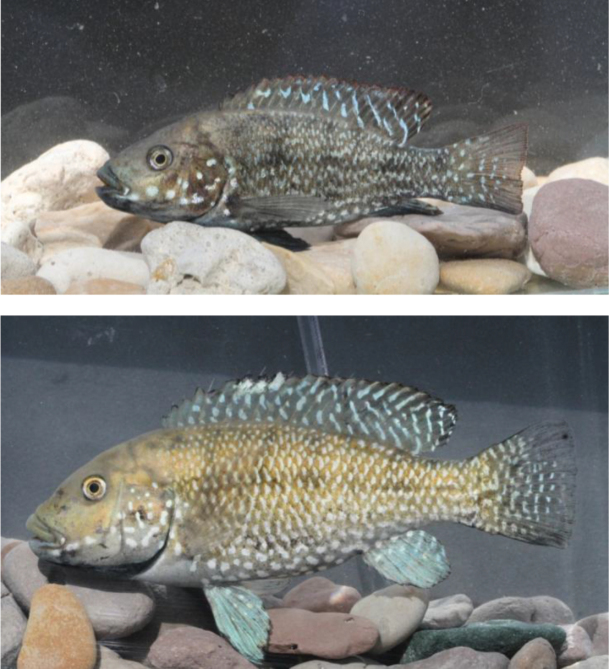
*Iranocichla* from Kol River drainage: ZM-CBSU IP25 male, 71.3 mm SL; Hormuzgan prov.: Faryab hot spring, Kol River drainage, ZM-CBSU-IP34 male, 112.9 mm SL; Hormuzgan prov.: Gode-Gaz Spring, Kol River drainage.

**Figure 11.**
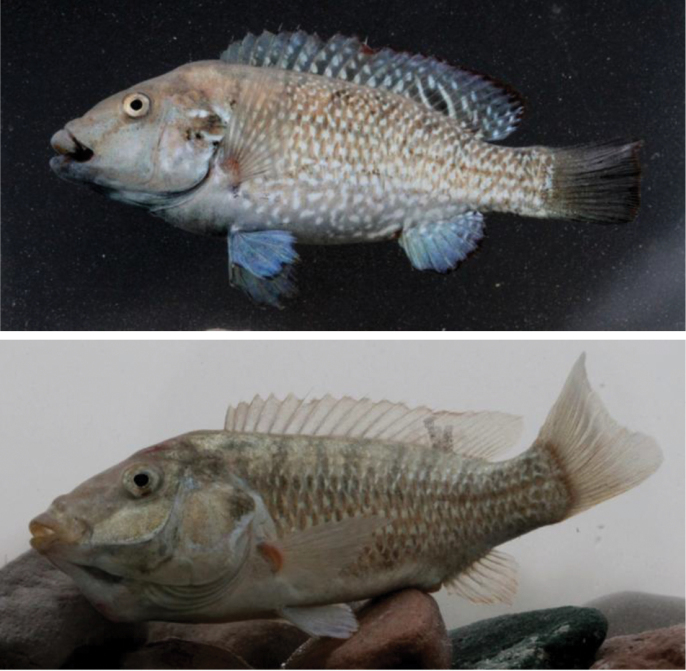
*Iranocichla hormuzensis*, ZM-CBSU-IH54 male, 93.6 mm SL, ZM-CBSU-IH50 female with eggs, 83.4 mm SL Hormuzgan prov.: Dezhgan, Mehran River.

The Kol River drainage is situated geographically between the Mehran River drainage, inhabited by *Iranocichla hormuzensis*, and the Shur River drainage, inhabited by *Iranocichla persa* (Fig. 1). The Kol populations (Figs 4, 10) show some morphological characters resembling *Iranocichla hormuzensis* and others resembling *Iranocichla persa*. Nuptial males from the Kol River drainage resemble *Iranocichla hormuzensis* in having a black breast and lower part of the head (vs. orange in *Iranocichla persa*). On the other hand, nuptial males from the Kol River drainage resemble *Iranocichla persa* in having only a much faded “*Tilapia*-mark” in the dorsal fin or none at all (vs. bold in *Iranocichla hormuzensis*) (see Figs 3, 4, 10, 11), and in having very clear white spots making almost wavy bars or stripes on the caudal fin (vs. without or with very few white spots in *Iranocichla hormuzensis*) (Figs 4, 11). There is one exception, these are fishes from the Faryab hot spring, which is a quite isolated small spring situated in the upper most reaches of the Kol River drainage (Fig. 1). In the Faryab hot spring, males resemble the nuptial coloration of those from Mehran River, albeit being nearly black with iridescent blue spots on caudal and dorsal fin being connected to stripes. However, our two males in breeding dress from this hot spring were smaller than those sampled from any of our other sites and larger male individuals from Faryab are needed to rule out an ontogenetic effect on the presence of “Tilapia-mark”. The nuptial males from the Shur and Minab River drainages have an orange edge to the dorsal fin. Nearly no differences in individual morphometric and meristic characters were found between the populations from the Kol and those from the Shur and Minab River drainages (Tables 1–3). The Kol populations resemble the latter and differ from *Iranocichla hormuzensis* in having a slightly more decurved dorsal head profile and a less pointed snout (Fig. 4).

**Table 2.** Morphometric and meristic data of *Iranocichla* sp. from Kol River drainage (Gode-Gaz stream) (ZM-CBSU34-40, H1547-H1551; n = 12).

	males (n = 7)				females (n = 5)			
	Min	Max	Mean	SD	Min	Max	Mean	SD
Standard length (mm)	83.4	109	95.2		59.6	69.7	63.0	
In percentage of standard length								
Head length	35.0	36.3	35.6	0.5	37.4	38.7	38.1	0.6
Pre dorsal length	35.6	37.4	36.3	0.8	38.5	40.2	39.6	0.8
Post dorsal length	36.1	40.0	38.6	1.5	36.3	39.5	38.3	1.3
Dorsal fin length	51.9	56.7	53.4	1.6	50.2	53.5	51.1	1.3
Anal fin length	10.6	12.2	11.6	0.5	8.8	11.1	10.0	0.9
Pre-anal length	73.1	77.4	74.9	1.6	75.3	76.9	75.8	0.6
Pectoral fin length	26.5	30.8	27.9	1.4	28.6	30.0	29.5	0.6
Pelvic fin length	21.2	23.7	22.3	0.9	18.8	22.2	20.5	1.4
Pre-pelvic length	38.6	41.7	40.4	1.1	40.3	42.2	41.1	0.7
Maximum body depth	35.4	37.0	36.3	0.6	34.1	36.4	35.6	1.0
Body depth at dorsal fin origin	34.2	35.8	35.2	0.6	33.0	34.7	34.0	0.7
Minimum body depth	13.2	14.0	13.5	0.3	11.5	13.4	12.4	0.7
Distance between P&V	13.7	15.5	14.3	0.6	12.1	13.7	12.8	0.6
Distance between V&A	35.6	40.1	37.6	1.5	36.5	39.4	38.1	1.3
Caudal fin length	23.2	25.9	24.8	0.9	25.9	27.1	26.3	0.4
Caudal peduncle length	16.7	18.1	17.3	0.5	16.7	18.3	17.4	0.7
In percentage of head length								
Head depth	83.8	87.4	85.3	1.2	77.4	90.7	83.4	5.0
Head width	50.9	53.9	52.3	1.1	49.9	52.8	51.8	1.1
Preorbital distance	38.8	43.5	41.5	1.6	39.9	40.5	40.2	0.2
Postorbital distance	44.2	47.1	45.6	1.1	42.9	44.0	43.3	0.4
Interorbital distance	28.6	30.8	29.8	0.9	31.2	31.8	31.7	0.3
Eye diameter	17.2	20.5	18.4	1.0	17.8	18.8	18.4	0.4
Meristic characters								
Scales in upper lateral line	17	23	20.6	2.1	17	21	19	1.6
Scales in lower lateral line	9	12	11	1.0	10	12	11.4	0.9
Dorsal fin unbranched rays	15	16	15.6	0.5	15	16	15.4	0.5
Dorsal fin branched rays	9	10	9.3	0.5	9	10	9.4	0.5
Anal fin unbranched rays	3	3	3	0.0	3	3	3	0.0
Anal fin branched rays	5	7	6.1	0.9	6	7	6.6	0.5
Pelvic fin unbranched rays	1	1	1	0.0	1	1	1	0.0
Pelvic fin branched rays	5	5	5	0.0	5	5	5	0.0
Pectoral fin rays	11	12	11.3	0.5	11	12	11.4	0.5
Gill rakers	14	16	14.9	0.9	14	16	15.4	0.9

**Table 3.** Morphometric and meristic data of *Iranocichla hormuzensis* from Mehran River (ZM-CBSU K1128-K1143, IH2-IH7, n = 22).

	males (n = 10)				females (n = 12)			
	Min	Max	Mean	SD	Min	Max	Mean	SD
Standard length (mm)	82.3	100.6	91.8	5.2	59.0	84.4	72.3	7.3
In percentage of standard length								
Head length	34.3	36.8	35.9	0.7	35.3	37.0	36.3	0.5
Pre dorsal length	35.7	39.3	38.0	1.1	38.1	40.1	39.0	0.6
Post dorsal length	34.0	37.6	35.3	1.0	32.8	36.3	34.7	0.8
Dorsal length	48.2	52.6	51.2	1.5	47.6	49.8	48.8	0.6
Anal length	9.1	12.2	10.7	0.9	9.4	10.3	9.8	0.3
Pre Anal length	69.2	72.2	71.1	0.8	71.1	73.3	72.7	0.6
Pectoral fin length	22.7	27.7	26.8	1.5	24.9	26.8	25.8	0.7
Pelvic fin length	16.3	19.9	18.4	1.1	15.9	18.1	17.3	0.6
Pre Pelvic length	38.9	41.5	40.0	0.9	40.7	43.6	41.9	0.7
Maximum body depth	32.3	39.2	35.7	1.8	31.9	37.5	34.5	1.4
Body depth at dorsal fin origin	31.7	36.9	33.9	1.5	31.5	34.4	33.0	0.9
Minimum body depth	13.0	14.2	13.6	0.4	12.0	13.2	12.5	0.4
Distance between P&V	12.8	15.5	13.7	0.9	12.4	13.8	12.9	0.4
Distance between V&A	30.6	34.6	33.1	1.2	31.3	33.6	32.5	0.8
Caudal fin length	21.9	23.9	23.1	0.6	21.7	25.1	23.0	0.9
Caudal peduncle length	19.4	21.2	20.2	0.6	19.3	22.0	20.5	0.9
In percentage of head length								
Head depth	60.2	69.2	65.2	2.9	64.4	72.4	69.1	2.3
Head width	48.1	59.0	53.7	3.5	52.3	61.6	56.1	2.4
Preorbital distance	40.0	43.1	42.1	0.9	41.2	43.9	42.3	0.7
Postorbital distance	42.1	45.9	44.0	1.2	42.5	48.4	46.2	2.2
Interorbital distance	25.7	36.8	32.0	3.6	33.1	36.9	34.4	0.9
Eye diameter	17.3	20.8	18.4	1.1	15.6	20.0	17.5	1.3
Meristic characters								
Scales in upper lateral line	16	21	18.2	1.8	15.0	21.0	18.2	2.1
Scales in lower lateral line	9	14	11.9	1.7	10.0	13.0	11.7	1.1
Dorsal fin unbranched rays	15	16	15.2	0.4	15.0	16.0	15.2	0.4
Dorsal fin branched rays	9	11	10.2	0.6	9.0	10.0	9.8	0.4
Anal fin unbranched rays	3	3	3.0	0.0	3.0	3.0	3.0	0.0
Anal fin branched rays	5	7	6.0	0.9	5.0	7.0	6.1	0.8
Pelvic fin unbranched rays	1	1	1.0	0.0	1.0	1.0	1.0	0.0
Pelvic fin branched rays	5	5	5.0	0.0	5.0	5.0	5.0	0.0
Pectoral fin rays	12	12	12.0	0.0	12.0	12.0	12.0	0.0
Gill rakers	14	17	15.9	1.0	15.0	17.0	15.8	0.7

**Table 4.** Teeth formula of *Iranocichla*. 1 = unicuspid; 2 = bicuspid; 3 = tricuspid.

Species	ZM-CBSU Number	Locality	Sex	SL (mm)	Upper jaw teeth formula	Lower jaw teeth formula
*Iranocichla persa*	25	Khorgo	M	81	14(1)+26(2)	2(1)+17(2)+2(3)
*Iranocichla persa*	SEM	Khorgo	M	60.7	12(1)+34(2)+1(3)	19(2)+4(3)
*Iranocichla persa*	147	Ziyarat Ali	F	73.7	43(2)+1(3)	1(1)+23(2)
*Iranocichla persa*	148	Ziyarat Ali	M	94.8	25(1)+29(2)+1(3)	5(1)+18(2)
*Iranocichla* sp. Kol	3	Lar	M	90.3	9(1)+36(2)+3(3)	5(1)+5(2)+4(3)
*Iranocichla hormuzensis*	295	Bastak-Mehran	M	76.7	4(1)+27(2)+12(3)	3(1)+21(2)+1(3)
*Iranocichla hormuzensis*	325	Bastak-Mehran	F	72.4	41(2)+20(3)	26(2)
*Iranocichla hormuzensis*	394	Kokherd	F	80.5	14(1)+32(2)+1(3)	20(2)
*Iranocichla hormuzensis*	398	Kokherd	M	82	8(1)+26(2)+3(3)	17(2)+1(3)

From a genetic point of view, according to Schwarzer et al. (2016), who used ND2 and D-loop sequences, the western Kol River populations combined (Kol, Gode-Gaz, Faryab, Tange-Dalan) but also Gode-Gaz and Faryab each on its own, were genetically more diverse than *Iranocichla* populations of other drainages, having 15 different haplotypes, none of which was shared with any other drainage system and making two clades separated by a minimum of 4 mutations. All of them belonged to clade B of Schwarzer et al., but while one of the clades was unique to the Kol drainage populations, sharing a relatively recent common ancestral haplotype with the *Iranocichla persa* haplotype clade, the other Kol river clade was shared with *Iranocichla persa*, albeit with many distinct haplotypes. Based on these results, Schwarzer et al. (2016) concluded that the nearly complete lack of haplotype sharing between the rivers, combined with distinct differences in male nuptial coloration, suggests the existence of two younger allopatric species within Clade B (Kol, Shur and Minab River systems): a red headed species in Shur, Hasanlangi and Minab (described as *Iranocichla persa* here) and a dark black one in the Kol river. The divergence of these forms is more recent and the western Kol populations appear to be para- or polyphyletic assemblages in their mitochondrial genes, possibly suggesting an old stable population or a case of more recent secondary contact and admixture with gene flow from the Shur River into the Kol River. The eastern arm of the Kol river harbours only one of the two haplotype clades, but Schwarzer et al. (2016) had limited sampling from that river arm. Note that the persistence of two divergent haplotype clades only in the western arm of the Kol River does not seem to be associated with any obvious phenotypic polymorphism within these sites, hence we do not see evidence for more than one species in any one river.

### Comparative materials

**Specimens from Kol River drainage.** ZM-CBSU IP24, 10, 39–71 mm SL; ZM-CBSU k1144, 5, 30–58 mm SL; Hormuzgan prov.: Faryab Hot Spring at Faryabe-Sanguyeh village, approx. 30 km north of Bastak city, 27°26'01.0"N 54°16'43.0"E.—ZM-CBSU IP34, 11, 67–113 mm SL; Hormuzgan prov.: Gode-Gaz (Gowde-Gaz) stream approx. 15 km east of Bastak city at Gode- Gaz village, 27°17'28.8"N 54°29'20.7"E. —ZM-CBSU IP45, 1, 78 mm SL; Hormuzgan prov.: Tange-Dalan stream, 27°23'14.6"N 55°00'14.0"E. —ZM-CBSU IP79, 3, 89–100 mm SL; Fars prov.: Lar stream, approx. 25 km east of Lar city, 27°38'19.0"N 54°41'33.2"E. —ZM-CBSU IP82, 1, 87 mm SL; Iran: Hormuzgan prov.: Kol River approx. 15 km north of Bandare-pol, 27°07'19.5"N 55°44'55.4"E.

***Iranocichla hormuzensis*.** All from Iran: Hormuzgan prov.: ZM-CBSU IH2, 11, 73–102 mm SL; Mehran River at Kokherd, approx. 50 km south-east of Bastak, 27°04'50.1"N 54°28'24.4"E. — ZM-CBSU K1128, 8, 68–101 mm SL; Mehran River at Kokherd, approx. 50 km south-east of Bastak, 27°04'50.1"N 54°28'24.4"E. — FSJF 3467, 82 mm SL; ZM-CBSU IH13, 11, 59–89 mm SL; ZM-CBSU K1136, 8, 59–84 mm SL; Mehran River at Gotow (Gotab), approx. 20 km south-west of Bastak, 27°08'37.2"N 54°15'44.70"E. — ZM-CBSU-IH46, 12, 68–94 mm SL; Mehran River at Dezhgan, approx. 35 km west of Bandare-Khamir, 26°52'55.4"N 55°16'20.8"E.

### Material used for molecular COI analysis

**Specimens from Kol River drainage.** All from Iran: — ZM-CBSU-IH45, Tange-Dalan stream, 27°23'14.6"N 55°00'14.0"E (GenBank accession number: KY034448). —ZM-CBSU M803, M804; Kol River at Lar, 27°38'19.0"N 54°41'33.2"E (GenBank accession numbers: KY034446, KY034447).

***Iranocichla hormuzensis*.** All from Iran: ZM-CBSU M921, M922, M939, M943; Mehran River at Kokherd, 27°04'50.1"N 54°28'24.4"E (GenBank accession numbers: KY034431, KY034432, KY034433, KY034434).

### Comparative material from GenBank

*Oreochromis niloticus* FJ348104.1, *Oreochromis niloticus* KJ554049, *Oreochromis niloticus* KJ553958, *Oreochromis aureus* KJ553787, *Oreochromis aureus* KJ553805, *Sarotherodon galilaeus* HM882887.1, *Sarotherodon galilaeus* FJ348122.1, *Astatotilapia burtoni* EU888024, *Astatotilapia desfontanii* KJ553606, *Astatotilapia desfontanii* KJ553501, *Astatotilapia desfontanii* KJ553392, *Astatotilapia desfontanii* KJ553501.

## Discussion

Female and juvenile *Iranocichla persa*, *Iranocichla hormuzensis* and those from Kol River drainage are difficult to distinguish from each other based on morphology, and males are best distinguished when they are territorial and show their nuptial coloration. Outside the breeding period, adult males can be distinguished based on differences in the retention of the “Tilapia-mark”, the iridescent spotting of the fins and differences in head shape. Both species are distinguishable by multiple fixed substitution between their mitochondrial lineages, as seen in their ND2, D-loop and COI sequences, suggesting a relatively old divergence of ~160 and 318 kya years (Schwarzer et al. 2016) between two readily diagnosable species, *Iranocichla hormuzensis* and *Iranocichla persa*.

The populations from Kol River drainage including Lar, Faryab, Gode-Gaz (Rasoul), Tange- Dalan, Kol River itself, whose haplotypes are either nested in the *Iranocichla persa* mitochondrial clade, or very closely related to it, show some phenotypic trait mosaic between the two species. Hence, male breeding coloration differs remarkably between the species and these differences coincide with major drainage system differences (Fig. 1). Based on available and presented data including the haplotype network and demographic history reconstruction conducted by Schwarzer et al. (2016) , low genetic diversity and little haplotype sharing, those authors proposed two possible scenarios for allopatric speciation of *Iranocichla* between the major river drainages (clades A, B): (I) *Iranocichla* populations persisted throughout the Pleistocene in the Mehran and Kol River systems, but remained isolated ever since their first split ~160 and 318 kya. (II) Shur and Mehran River drainages (including Rudan River) were only colonized during or after the Last Glacial Maximum (LGM) from the Kol river drainage, most likely through occasional long distance dispersal through the Strait of Hormuz. Alternatively, *Iranocichla* of clade B may have entered the Shur River system earlier and persisted both in Shur and Kol River drainage during droughts in the LGM. They may then have colonized the Rudan River in the Minab River system from Shur and also recolonized the Kol River system from the Shur during or just after the LGM. This would explain the central position within clade B of Shur haplotypes in the haplotype network and the existence of and admixture between two haplotype clades in the western arm of the Kol River system. Yet another possibility is that Shur and Kol river systems were colonized at the same time (the reconstructed most recent common ancestor haplotype of all Kol, Shur, and Rudan haplotypes is extinct or absent in our data) and started to diverge into a western Kol and an eastern Kol/Shur clade, and that the western Kol afterward was colonized a second time from the east through river capture of the eastern Kol which may once have drained toward the Shur as indicated by the river topology of the eastern Kol (Fig. 1). Subsequently more recent bottlenecking of eastern Kol and Shur populations could have led to their divergence in haplotype frequencies without much sequence divergence (see Schwarzer et al. 2016). The exact taxonomic status of the Kol River populations awaits further research.

## Supplementary Material

XML Treatment for Iranocichla persa
